# Structural and functional identification of vasculogenic mimicry *in vitro*

**DOI:** 10.1038/s41598-017-07622-w

**Published:** 2017-08-01

**Authors:** Dusan Racordon, Andrés Valdivia, Gabriel Mingo, Rafaela Erices, Raúl Aravena, Felice Santoro, Maria Loreto Bravo, Carolina Ramirez, Pamela Gonzalez, Alejandra Sandoval, Alfonso González, Claudio Retamal, Marcelo J. Kogan, Sumie Kato, Mauricio A. Cuello, German Osorio, Francisco Nualart, Pedro Alvares, Araceli Gago-Arias, Daniella Fabri, Ignacio Espinoza, Beatriz Sanchez, Alejandro H. Corvalán, Mauricio P. Pinto, Gareth I. Owen

**Affiliations:** 1 Department of Physiology, Faculty of Biological Sciences, Pontificia Universidad Católica de Chile, Santiago, Chile; 2 Division of Obstetrics & Gynecology, Faculty of Medicine, Pontificia Universidad Católica de Chile, Santiago, Chile; 3 Universidad Santo Tomás, Santiago, Chile; 4 Biomedical Research Consortium of Chile, Santiago, Chile; 5 Millennium Institute on Immunology and Immunotherapy, Pontificia Universidad Católica de Chile, Santiago, Chile; 6 Department of Hematology and Oncology, Faculty of Medicine, Pontificia Universidad Católica de Chile, Santiago, Chile; 7 Center UC Investigation in Oncology at Pontificia Universidad Católica de Chile, Santiago, Chile; 8 Advanced Center for Chronic Diseases (ACCDiS), Faculty of Medicine, Pontificia Universidad Católica de Chile, Santiago, Chile; 9 Facultad de Medicina, Universidad San Sebastián, Santiago, Chile; 10 Facultad de Ciencias, Universidad San Sebastián, Santiago, Chile; 11 Centro de Envejecimiento y Regeneración, Facultad de Ciencias Biológicas, Pontificia Universidad Católica de Chile, Santiago, Chile; 12 Department of Cellular Biology, Faculty of Biological Sciences, Universidad de Concepcion, Concepción, Chile; 13 Institute of Physics, Pontificia Universidad Católica de Chile, Santiago, Chile

## Abstract

Vasculogenic mimicry (VM) describes a process by which cancer cells establish an alternative perfusion pathway in an endothelial cell-free manner. Despite its strong correlation with reduced patient survival, controversy still surrounds the existence of an *in vitro* model of VM. Furthermore, many studies that claim to demonstrate VM fail to provide solid evidence of true hollow channels, raising concerns as to whether actual VM is actually being examined. Herein, we provide a standardized *in vitro* assay that recreates the formation of functional hollow channels using ovarian cancer cell lines, cancer spheres and primary cultures derived from ovarian cancer ascites. X-ray microtomography 3D-reconstruction, fluorescence confocal microscopy and dye microinjection conclusively confirm the existence of functional glycoprotein-rich lined tubular structures *in vitro* and demonstrate that many of structures reported in the literature may not represent VM. This assay may be useful to design and test future VM-blocking anticancer therapies.

## Introduction

Cancer cells within a tumor require a blood supply containing oxygen and nutrients in order to grow beyond a few millimeters^1–3^. Growing tumors have the ability to stimulate the generation of new blood vessels from preexisting ones, a process called angiogenesis^1^. This is achieved by the synthesis and secretion of proangiogenic factors such as the Vascular Endothelial Growth factor (VEGF)^1^. Over the last decades the development of angiogenesis inhibitors such as Bevacizumab (an anti-VEGF humanized antibody) or tyrosine kinase inhibitors that target the VEGF pathway^4^ have improved cancer therapies for patients and today antiangiogenic drugs are widely used for the treatment of many solid tumors^4, 5^. However, it is well documented that in many cases a successful initial responseis followed by the acquisition of resistance to antiangiogenesis and eventually tumor recurrence^4^. Many strategies have been postulated to explain this phenomenon^5^. One of these strategies involves a phenotypic switch in certain cells within a tumor that modify their morphology to form a network of fluid-conducting tubular structures, establishing an angiogenesis-independent alternative perfusion pathway into the tumor, a process known as vasculogenic mimicry (VM)^6^.

Although it was originally described in uveal melanoma^7^, many studies have demonstrated VM in a variety of malignancies including skin melanoma, lung, gastric, and colorectal cancers^8^. However, certain authors have suggested VM might actually be a physiological mechanism, used by angioblasts (Endothelial cell (EC) precursors) during embryonic vasculogenesis (EV); in this process, precursors can differentiate and connect forming a “honeycomb-like network” of tubular structures^9, 10^ that recapitulates the morphology observed in VM. This notion is further supported by a recent study that demonstrates that macrophages can remodel to create VM channels^11^.

The ability of certain cancer cell subpopulations to undergo VM has been confirmed using both *in vivo* and *in vitro* models^7^. Tubular VM structures are indeed different from traditional blood vessels formed by angiogenesis that are comprised of ECs. In VM, tubular structures are formed by cancer cells that surround a matrix-rich sheet with a central lumen, without ECs^7, 12–15^. The functionality of VM tubular structures has been confirmed by tumor biopsies that demonstrate the presence of red blood cells in the lumen of these structures^7, 13, 16, 17^. Traditionally, the presence of VM in tumor biopsies is detected by Periodic Acid Schiff (PAS) staining of samples combined with the absence of specific EC markers^18^. In patients, the presence of VM in tumor biopsies is associated with poor overall survival^8, 13, 19, 20^. In stark contrast, *in vitro* studies on VM are far more controversial. Only a handful of studies provide evidence of hollow tube formation^7, 11, 13–15, 21–24^ or use cell lines previously confirmed to form tubular structures^10, 15, 25–27^. Many publications claim to observe VM based on the observation of cancer cell rearrangements and VM is simply assumed by a morphological criterion and its resemblance with tubular structures normally observed in traditional EC-based *in vitro* angiogenesis assays^28–32^. Here we sought to address this controversy by extensively characterizing VM *in vitro*, providing conclusive evidence that hollow tubes exist, they are lined by secreted glycoproteins and are indeed capable of conducting fluids.

Although VM has been recently observed in other cancer types^18, 33^ ovarian cancer was one of the first carcinomas in which VM was described, and shown to correlate with decreased overall survival^13, 19^. The main goal of our study was to characterize structurally and functionally the presence of VM tubules using ovarian cancer cells as a platform. We demonstrate using ovarian cancer cell lines, ovarian cancer cell spheres (a surrogate of metastasis-initiating cells) and patient samples the ability of cancer cells to form VM *in vitro*, confirmed by dye microinjection and X-ray microtomography 3D-reconstruction and confocal microscopy. We demonstrate herein that tubular structures are present in *in vitro* cultures and are capable of conducting fluids.

## Results and Discussion

### An *in vitro* assay for VM in ovarian cancer cells

**Figure 1:**
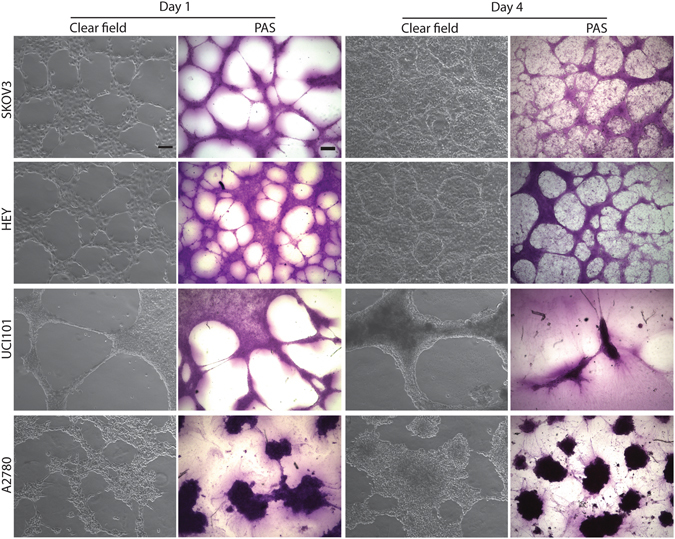
Ovarian cancer cell lines form tubular structures in 3D culture. Clear field images (magnification: 10×. Scale bar: 100 μm) and PAS stained images (magnification: 4×. Scale bar: 200 μm) of 3D cultures on matrigel at day 1 (left panels) and day 4 (right panels) in SKOV3, HEY, UCI101 and A2780 cells. At day 1 all cell lines form structures that could be described as capillary-like and are PAS+, however at day 4 only SKOV3 and HEY cell lines form cell aggregates surrounded by capillary-like structures. Tubular structures in SKOV3 and HEY were more PAS+ compared to the monolayer of cells, however in UCI101 and A2780, the individual cells stained strongly for PAS.

Ever since its initial report in 1999 VM ignited a vigorous debate in the scientific community^8, 34, 35^. During the following years clinical studies unequivocally demonstrated the existence of VM in human tumor biopsies and in *in vivo* experimental settings using PAS staining along with an absence of EC markers in blood containing vessel-like structures within tumors^16, 18^. On the other hand, *in vitro* VM studies are less conclusive^7, 13–15, 21, 22, 24^ and so far they have failed to provide a definitive proof of a lumen in vessel-like structures. Therefore, in order to establish an *in vitro* method to assess VM, we first set up starting cell numbers and culture times required to observe VM *in vitro*. Using four ovarian cancer cell lines (SKOV3, HEY, UCI101 & A2780, Fig. 1), first we determined the optimal cell numbers needed to obtain tubular structures similar to those previously reported by several authors^7, 13, 14^. Notably, all cell lines formed structures that could be described as capillary-like starting at day 1 in culture, and as described by many authors, they resembled the structures observed in traditional ECs assays (Supplementary Fig. 1a–d, day 1:15,000 and 75,000 cells)^28, 36, 37^. After three days cells cover most of the culture area, and in SKOV3 and HEY there is the appearance of circular cell aggregates or the appearance of a monolayer below potential tubular structures. At day 4 structures mature (thicken) to form notable channels and spread across the plate with a diameter in the 50 to 150 μm range (Supplementary Fig. 1a,b). These structures now appear to be at a higher plane compared to the general cell population of cancer cells, an effect that is more evident at day 7. From day 7 to day 10 there is a further increase in diameter and now a notable cellular detachment. Furthermore, in the two cell lines that formed tubular structures (SKOV3 and HEY) PAS staining was predominantly present along the length of these structures. However in UCI101 and A2780 there was uniformly strong staining of PAS within the cell structures.

In respect to cell number, in SKOV3 there is a formation of tubular structures at day 4 using 15,000, 75,000 and 150,000 starting cell number. At 300,000 cells there was formation at day 4, but a loss of tubular structures at day 10. At 750,000 cells we did not observe a clear formation of tubular structures (Supplementary Fig. 1a). This pattern was generally repeated in HEY, with the exception of day 10 with 150,000 cells where there was evident detachment of cells (Supplementary Fig. 1b). In conclusion, 15,000 and 75,000 starting cell numbers gave the best results, with clear tubular formation at day 4.

Overall, the morphology using optimal starting cell numbers required to obtain *in vitro* tubular structures seems to be cell-specific and is shown in Fig. 1 (left panels). VM structures are reported to be rich in extracellular matrix, including proteoglycans and glycoprotein-like laminin, and are therefore PAS+^7, 12^.

**Figure 2:**
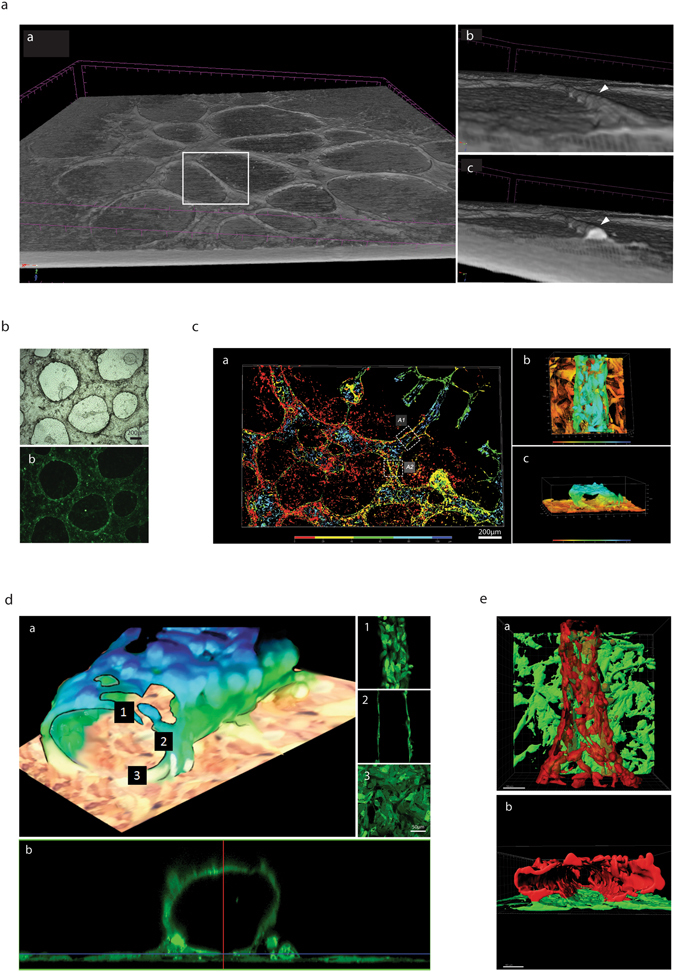
Lumen–containing tubular structures are present in ovarian cancer cell lines in culture. (**a**) 3D-reconstruction of X-ray microtomography: panel a, Reconstructed view of the landscape of a 4 day 3D-culture of SKOV3 cells. Elevated structures with tubular-like appearances are clearly visible. The structures within the white rectangle are shown in higher magnification in panel b and c, with the arrowhead denoting a seemingly tubular structure projecting above the flat cell aggregates. A cross-section of this structure is shown to demonstrate an air-filled space (panel a,c) with an estimated diameter of 50 μm. (**b**) 4 day-old 3D-culture of GFP-HEY cells as demonstrated in clear field (**a**) and fluorescence (**b**). (**c**) Confocal 3D reconstruction of 4 day-old 3D-culture of GFP-Hey cells with the color map discriminating between the planes (height of structures) as indicated in the key. (**b**) Amplification of region A1 demonstrating the presence of a cell-containing structure in a higher plane respect to the monolayer of cells. When rotated, same image reveals the presence of a lumen (**c**). (**d**) A confocal microscopy Z-stack reconstruction of region A1 demonstrating the presence of a cell containing tubular structure. Z-Stack demonstrates a continuous upper monolayer (1), with central walled structures with a hollow center (2) and a continuous lower monolayer (3). This is also shown clearly in the computer-generated cross-section in panel b. (**e**). Imaris software reconstruction of area A2 from panel c, showing cancer cells in two distinct planes, the red plane being elevated in respect to the lower green plane. A 90° backward rotation of this structure clearly shows the presence of a lumen (**b**). This representation, that exclusively shows cancer cell fluorescence, demonstrates a fenestrated appearance. Please also refer to Supplementary Video 2.

To confirm the presence of VM it is necessary to demonstrate the existence of a lumen within a tubular structure. To this end we performed X-ray microtomography (microCT) analysis^38–40^ upon structures formed after 4 days in matrigel cultures (Fig. 2a). Analysis shows that ovarian cancer cells organize on clusters (cell aggregates) that are clearly distinguished from the surrounding tube-like structures that project above them (Fig. 2a and Supplementary Video 1). Not all elevated structures, which correspond to PAS+ stained, appear to present a lumen and may simply represent glycoprotein-rich areas. Across section of the 3D reconstructed microCT analysis shows that numerous structures (especially the structures that look tubular) contain a central region with lower intensity value (arrowhead, Fig. 2a, panel c and Supplementary Fig. 3). These lumen-containing structures possess an average diameter of 50–100 μm.

To further confirm the presence of a lumen green fluorescent protein (GFP)-labeled HEY ovarian cancer cells (Fig. 2b) were analyzed by confocal microscopy. Reconstruction (Fig. 2c) confirms that tubular structures are located on top of a layer of cells and are usually surrounded by relatively flat cell clusters (aggregates), as previously shown by microCT. Figure 2d shows a Z-stack analysis and a cross section view of a single tube; right panels show upper, middle and lower sections of a tubule (Fig. 2d, 1–3), additionally a side view confirms the presence of a lumen (Fig. 2d, panel b). A second reconstruction shows that tubules are fenestrated with a lumen diameter that ranges from 50 to 200 μm again consistent with our microCT data (Fig. 2a). A view from the top and a cross section are shown in Fig. 2e. Micro CT and confocal microscopy reconstruction videos can be viewed as supplementary material (Supplementary Videos 1 and 2).

**Figure 3:**
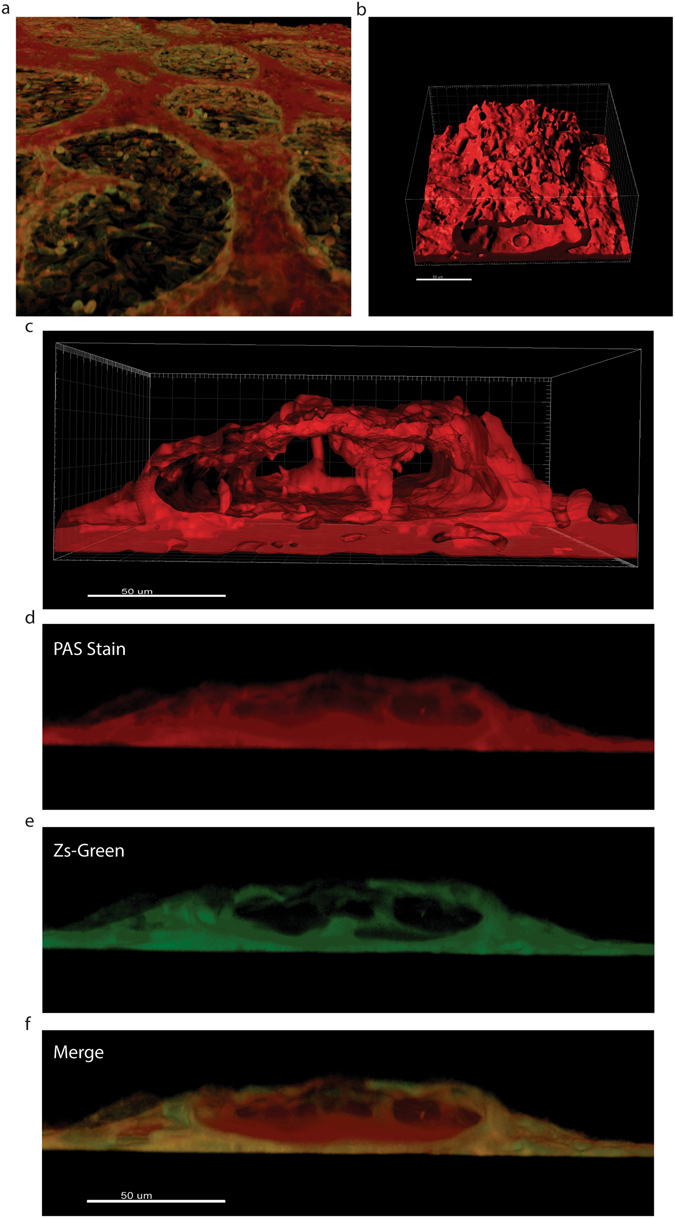
Tubular Structures possess a glycoprotein-rich internal layer. (**a**) 3D reconstruction of the monolayer and tubular structures formed by the HEY-GFP ovarian cancer line upon PAS staining and analysis with laser confocal microscopy. PAS emits within the red spectrum upon laser excitation at 560 nm. (**b**) Reconstruction of the glyoprotein-rich component of a tubular structure. (**c**) Reconstruction of the glyoprotein-rich component surrounding a lumen. (**d**,**e**) Cross-section of PAS stained tubular structures. In red (panel d) a glycoprotein-rich component constructed from PAS staining. In green (panel e) cancer cells forming a tubular structure, (**f**) merge of the previous panels showing that the glycoprotein-rich component is present on luminal side of the green (cancer cell) tubular structures.

Reconstruction demonstrates that ovarian cancer cells organize into tubular shapes that appear to be highly fenestrated, however the fluorescent image refers only to the GFP-labeled cancer cells. This suggests that there is in fact a matrix secreted by the cancer cells that lines the lumen, in accordance with the presence of a strong staining for carbohydrates (PAS+, Fig. 1). To test this hypothesis we returned to the technique of confocal microscopy and took advantage of the observation that PAS stained structures fluoresce when stimulation by laser at 560 nm. As can be shown in Fig. 3a, PAS staining takes the form of a tubular structure. Furthermore, reconstruction with the fluorescent cancer cells demonstrates that this is a layer of dense glycoprotein accumulation occurs between the lumen and the layer of cancer cells (Panels B–F in Fig. 3). A video (Supplementary Video 3) constructed by the IMARIS confocal software further shows evidence of an internal glycoprotein-rich layer.

**Figure 4:**
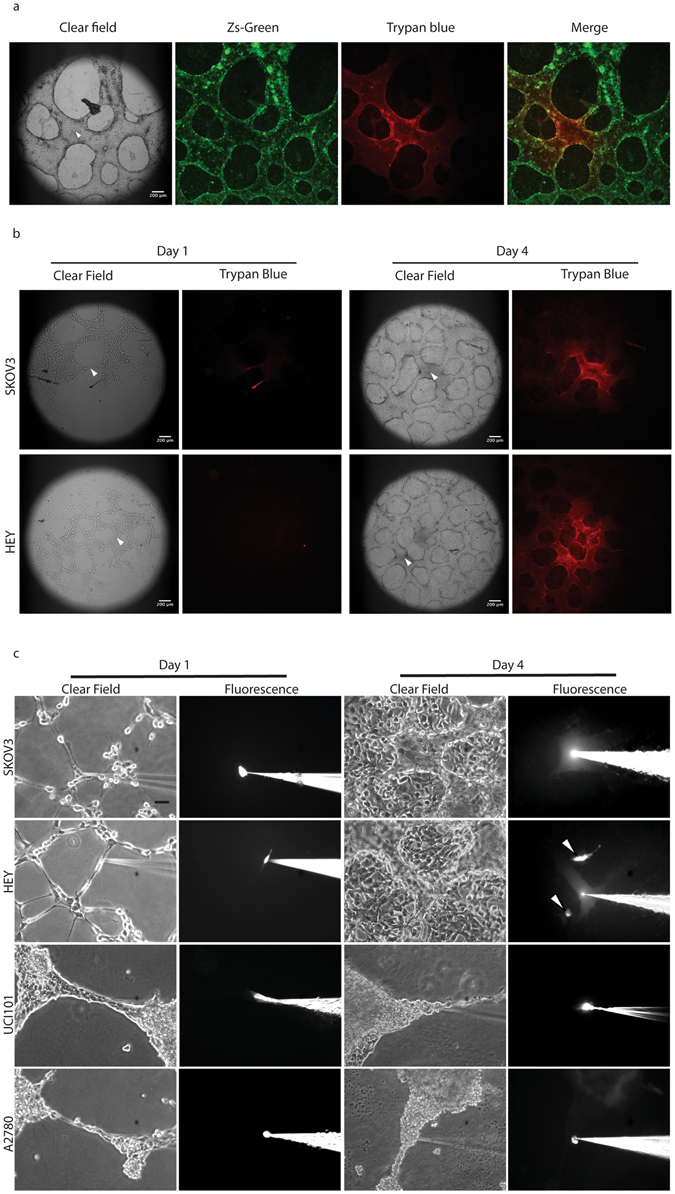
*In vitro* tubular structures are capable of fluid conduction. (**a**) Trypan blue was micro-injected under constant pressure during 20 minutes into fixed 4 days-old GFP-HEY cell cultures. The arrowhead indicates the site of injection into the tubular network. (**b**) 3D-cultures of parental SKOV3 and HEY cells were fixed at days 1 (left) and 4 (right) and trypan blue microinjection was performed. Dye movement was limited to structures formed at day 4 in both cell lines. Arrowhead indicates the site of microinjection. (**c**) Lucifer Yellow Dye (LYD) microinjection in living-cells SKOV3 and HEY cultures at day 1 (left) and 4 (right). No dye conduction was observed at day 1 under any condition. At day 4, LYD diffused along tubular structures surrounding clusters of cancer cells. Arrowheads indicate cells (within clusters between the tubular structures) microinjected with LYD, demonstrating that fluid could not pass from cell to cell. In UCI101 and A2780 (lower panels) no dye conduction was observed at day 1 (left) or 4 (right). Magnification: 10×. Scale bar: 100 μm.

Having established the presence of a lumen that is coated by a carbohydrate matrix and then by cancer cells, the next step was to confirm functionality. To achieve this we used a microinjector to allow the application of trypan blue under constant pressure in GFP-labeled HEY cells (Fig. 4a). The suspected tubular structures were indeed capable of conducting the dye (which naturally appears as a red fluorescence) with no obvious leakage into the surrounding structures. These experiments were also performed using parental unlabeled cell lines (SKOV3 & HEY, Fig. 4b) with similar results at Day 4 in culture. Importantly, fluid microinjection experiments were performed on fixed cultures, which rules out any possible contribution of dye movement as a result of cells coupled via gap junctions. Furthermore, this also facilitates VM analysis, as the assays can be fixed and stored for up to a week prior to microinjection procedures.

While we clearly demonstrate that the 50–200 μm structures present at day 3–4 in culture contain a lumen and can effectively move a dye, doubt remains over structures that appear to have intercellular connects between individual cancer cells at day 1 in culture on matrigel. To address functionality of potential tubular structures we performed microinjections at Day 1 and 4 with Lucifer Yellow Dye (LYD) in a panel of ovarian cancer cell lines (Fig. 4c). Our results confirm that SKOV3 and HEY form functional tubules at Day 4 in culture (upper panels). In contrast, microinjections at day 4 in UCI101 or A2780, or in any structures formed at day 1 in each of the four cell lines tested failed to demonstrate the movement of LYD (lower panels). These experiments were performed in living cell cultures (non-fixed) and thus cellular coupling is theoretically intact. Interestingly, we have noticed that the formation of intercellular connections at day 1 is not a prerequisite of later fluid-conducting tubular structures, despite the cell line in question being capable of forming VM structures at day 3–4.

Overall, our results demonstrate that a functional test such as fluid microinjection is absolutely required in order to discriminate between cell arrangements (capillary-like structures) and true hollow functional channels when using an *in vitro* assay^7, 14, 22, 24^. The microCT experiments suggest that many PAS+ areas (the current standard method to quantify VM in tumors) do not display a lumen and therefore cannot be considered VM when using *in vitro* assays. Despite this, we reaffirm the work of others that have established the basic criteria for VM *in vitro*: first, formation must be EC-free. Second, tubular structures must be PAS+ and finally, fluid conduction must be observed along the structure^7^.

### Primary cultures from ascites and cancer spheres also undergo VM *in vitro*

**Figure 5:**
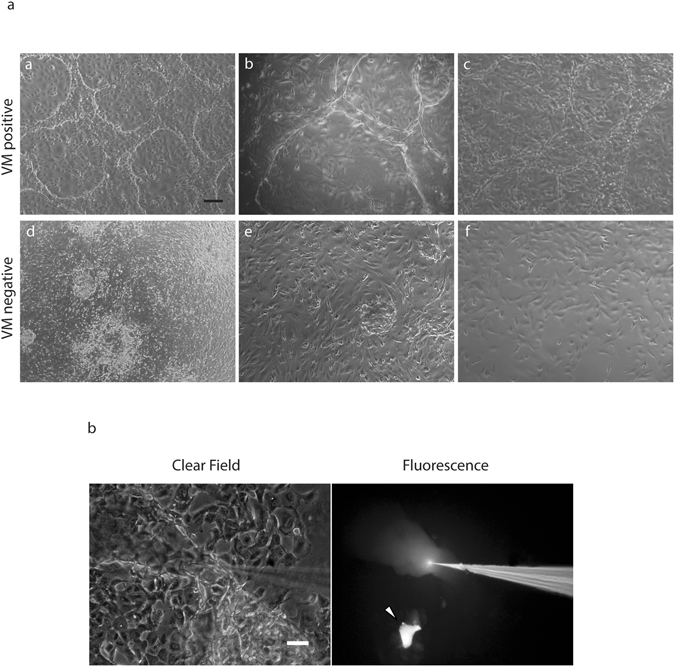
Tubular structure formation in primary cultures derived from ovarian cancer patient ascites. (**a**) examples of tubular structure formation in primary cultures derived from ovarian cancer patients ascites at day 7 (**a**–**c**). For comparison, three cultures that did not demonstrate VM formation are shown (**d**–**f**) Magnification: 4×. Scale bar: 200 μm. (**b**) LYD microinjection into a tubular structure formed in 3D-culture primary culture on matrigel. The arrowhead indicates microinjection into an individual cell, demonstrating no dye movement. Magnification: 10×. Scale bar: 100 μm.

**Figure 6:**
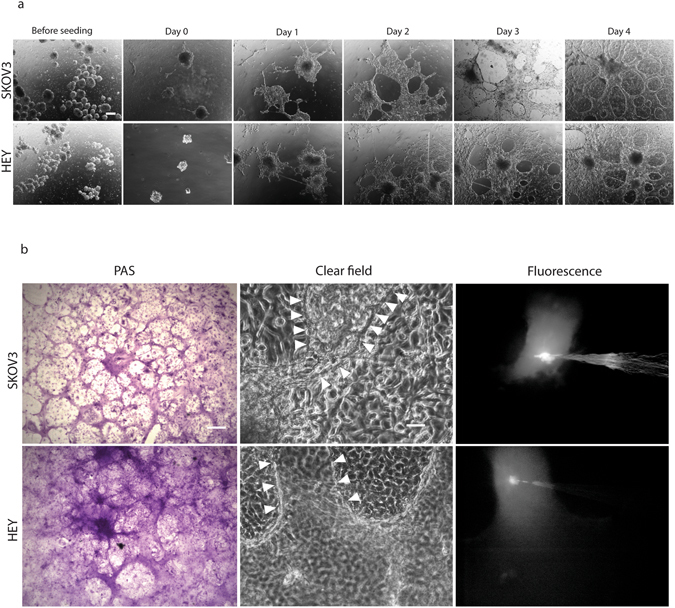
Tubular structure formation in ovarian cancer cell-derived spheroids. (**a**) Cancer spheres were generated from parental SKOV3 and HEY cells (noted as “before seeding”) and individual spheres seeded onto matrigel. At day 1 the cells grew principally within the spheres structure before spreading out to cover the dish at day 3 and forming clear tubular structures at day 4 in both cell lines. Magnification: 4×. Scalebar: 200 μm. (**b**) PAS staining (left) and microinjection (middle and right) of a 4 day-old spheres initiated 3D-cultures on matrigel. Arrowheads line the borders of microinjected structure. Magnification: 10×. Scalebar: 100 μm.

Ovarian cancers metastasize when cells shed from the primary tumor and disseminate into the peritoneal cavity, a process facilitated by the accumulation of intra peritoneal fluid, called ascites^41^. We have previously demonstrated the isolation and culture of ascites-derived ovarian cancer cells extracted from ovarian cancer patients^42, 43^. To date, our results show that 5/13 or 38.5% (Supplementary Table 1) of ascites isolated primary cultures assayed were capable of forming VM structures. The morphology of VM+ and VM− patient samples is shown in Fig. 5, VM+ samples were also functional (Fig. 5b). This demonstrates that not all cancer cell cultures are capable of undergoing VM and this is in line with previous pathology-based reports showing that VM is present in only 43% of ovarian tumor biopsies^44^. As pointed out, VM is not exclusive for cancer cells and this result supports this idea, and demonstrates that VM can be recapitulated in primary cultures and in a frequency similar to that observed in primary tumors. While we show that populations of ovarian cancer cells from ascites can be cultured and undergo VM, the cancer cells in the patient ascitic fluid survive in the form of cell aggregates (called spheres). It is believed that cancer spheres eventually adhere to the peritoneal walls with the establishment of a metastatic foci^45, 46^. Cancer spheres can be obtained *in vitro* by culturing cancer cell lines or primary cultures over non-adherent surfaces^43, 47^. Therefore next, we obtained cancer spheres from ovarian cancer cell lines^43^ and evaluated the ability of spheres to form VM structures (Fig. 6a). After seeding onto matrigel, the cancer spheres grew for 24 hours as enclosed circular structures, before expanding to cover the culture dish between days 3–4. Similar to our previous experiments, cancer sphere derived cell cultures were PAS+ and functional at Day 4 (Fig. 6b). To the best of our knowledge this is the first demonstration of VM *in vitro* originating from cancer sphere cultures.

As explained, cancer spheres serve as an *in vitro* model of metastasis initiating cells; growing ovarian cancer cells over non-adherent surfaces induces a switch to a mesenchymal phenotype via an epithelial-to-mesenchymal transition (EMT). In keeping with our observation that individual cancer spheres can expand to form tubular structures, the process of EMT has been shown to be a critical requirement for VM in cell cultures that report VM structures similar to those previously confirmed to conduct fluid in our experiments^48^. Since both SKOV3 and HEY cancer spheres form functional PAS+ tubular structures we can speculate that the ability to form VM structures is not a particularity of one cell line, or explained by a cell line derivation. The validation of our assay using ovarian cancer patient samples suggests that the ability of a subset of tumor cells to undergo VM is retained *in vitro* and therefore opens the possibility of using our assay as an outcome predictor and to evaluate potential anti-VM targeted treatments. Interestingly, our results show that cells from a patient with pathology-confirmed borderline ovarian cancer can also undergo VM (Fig. 6b and Supplementary Table 1), suggesting again that VM is not exclusive for advance stage cancers and may be present from early stages of cancer progression.

Future studies using our assay should expand into defining the contribution of specific VM signaling pathways. In line with a previously published report, we confirm using our assay conditions that PhosphoInositide 3 Kinase (PI3K) inhibition decreases VM formation*in vitro*, despite the culture being able to grow and reach confluence (Supplementary Fig. 2)^27^.

## Significance/Conclusions

VM is a poor prognosis factor present in many malignancies and thus a confirmed and standardized *in vitro* model is essential in order to study this phenomenon and convert it into a “druggable” target. Here we provide a standardized *in vitro* assay and a structural and functional characterization of VM in ovarian cancer cells. We present the most convincing evidence to date that VM can be reproduced in cell culture. Furthermore, we demonstrate that VM channels possess an inner layer of glycoproteins surrounded by cancer cells. The assay can be used as a platform in a variety of cell types including patient samples. Our results show that tubular structures *in vitro* are generally formed over a layer of cells. When grown on matrigel, most cancer cells form cell-cell connections or capillary-like structures within 24 hours in culture. Our experiments show that in some cases these capillary-like structures eventually become functional tubules (Fig. 2a,b and c), however in certain cells structures remain but are not functional (UCI101 and A2780, Fig. 3b). The formation of VM structures *in vitro* seems to be cell-specific; this is a feature that is maintained even in patient samples and at present it is unclear which are the cell determinants required for VM+ *in vitro* or *in vivo*. Finally, PAS positivity is often considered the standard to quantify VM. We recognize PAS is a useful tool in tumor samples or in *in vivo* experimental settings, however *in vitro* we demonstrate by microCT that large PAS+ areas are lacking a lumen (Fig. 2a) and are likely to be non-functional. Furthermore, we demonstrate in Fig. 1 that certain cancer cells are intensely stained with PAS (UCI101 & A2780, lower panels), however these tubular structures are not functional (Fig. 3c). Therefore, quantification based on PAS staining should be treated cautiously and confirmation of VM presence must be complemented with a functional test such as trypan blue or LYD microinjections. Herein we present conclusive evidence that tubular structures that are capable of conducting fluids can exist in culture. Furthermore, in contrast to vascular structures (blood vessels) where the endothelial cells are next to the lumen with a rich glycoprotein-containing basal lamina situated below, the vasculogenic mimicry structures presented in this paper contain an inner layer of yet to identified glycoprotein-rich structure next to the lumen with a cancer cell layer being situated below.

## Materials and Methods

### Reagents

Cell culture reagents including: RPMI 1640 (Cat. N°. 23400–021), DMEM-F12 (Cat. N°. 12400–024), fetal bovine serum (FBS, Cat. N°. 10437–028), penicillin/streptomycin (Cat. N°. 15240–062), Epidermal Growth Factor (EGF, PH50313) and insulin (Cat. N°. 12585–014) were from Gibco Life Technologies (Ontario, Canada). Fibroblast Growth Factor (FGF, Cat. N°. AA10–155) was from Invitrogen (Carlsbad, CA). Bovine serum albumin (BSA, Cat. N°. LY0087) was from Boval (Cleburne, TX). Ethanol (Cat. N°. 1.00983.2500), periodic acid (Cat. N°. 1359006), Schiff reagent (1.09033.0500) and aquatex (1.08562.00500) were purchased from Merck (Darmstadt, Germany). Sodium metabisulphite (Cat. N°. S9000) was purchased from Sigma-Aldrich (St. Louis, MO). Growth factor reduced, phenol red-free Matrigel (Cat. N°. 356231, here after called Matrigel) was purchased from Corning (Bedford, MA). LY294002 (Cat. N°. sc-215273) was purchased from Santa Cruz Biotechnology (Dallas, TX). Formaldehyde (Cat. N°. BM-0780) and hydrochloric acid (Cat. N°. BM-0065) were purchased from Winkler Ltda (Santiago, Chile).

### Cell Lines

SKOV3, HEY, and UCI101 were obtained from ATCC (Manassas, VA); A2780 were purchased from Sigma-Aldrich (St. Louis, MO)^49–52^. GFP-HEY (ZsGreen) cells were generated as described^53, 54^. All cells were routinely passaged in RPMI 1640 15% FBS and 1% penicillin/streptomycin. All cell lines are continually assessed for mycoplasma. Cells were maintained in a humidified incubator at 37 °C in 5% CO_2_. Experiments used only authenticated low passaged cell lines.

### Patient Samples

Human ovarian cancer ascites samples were obtained from multiple health institutions, with prior written informed consent obtained from all patients. All experiments were performed in accordance with the Declaration of Helsinki and the research protocol was approved by the ethics committee of each participating institution and health board: Faculty of Medicine at the Pontifical Catholic University of Chile; Foundation Arturo Lopez Perez Cancer, South Eastern & Eastern Metropolitan Medical Services (SSMSO & SSMO, Santiago de Chile) and the Quillota Medical Service (Region V, Chile). Primary cultures from ovarian cancer ascites and from washings of the peritoneal cavity were established as previously described by our group and others^43^.

### VM *in vitro* Assay: Three dimensional (3D) cultures

Experiments were performed with 70–80% confluent cell cultures. 3D cultures were performed as follow: 18 × 18 mm glass coverslips (Cat. N°. 0101030, Marienfeld, Lauda-Königshofen, Germany) were ethanol-washed, air-dried and placed in 6-well culture plates (Cat. N°. 140675, Thermo Scientific, Waltham, MA), coated with 50 μL of Matrigel per coverslip and air-dried for 45–60 min at room temperature. Cell cultures were trypsinized, counted and each cell number was resuspended in 200 μL of culture medium, that was seeded on matrigel-coated coverslips. Cells were incubated at 37 °C for 1 hour to allow its adhesion to the matrix and then covered with 3 ml of culture medium. Fresh medium was added every 2 days. For experiments with LY294002, culture medium was changed daily.

### PAS staining

3D-cultures were fixed in 4% formaldehyde in Phosphate Buffered Solution (PBS) 1X for 30 minutes at room temperature, then quickly washed with 1X PBS and 1X Tris Buffered Solution (TBS) for 15 min. Coverslips were incubated with 0.5% periodic acid for 10 min, washed with distilled water for 10 min and treated with Schiff reagent for 25 minutes. After washing with distilled water for 15 minutes, cultures were treated with a aqueous solution of 0.05 N HCl and sodium metabisulphite 5 mg/mL for 15 min. Stained cultures were then washed with distilled water for 15 minutes and mounted with aquatex for microscopy.

### Cancer Spheres 3D cultures

For sphere 3D-cultures, 200,000 cells of each cell line were seeded on ultra low attachment surface 6-well plates (Cat. N°. 3471, Corning, Bedford, MA) in CIC medium (DMEM-F12 supplemented with 10 ng/mL FGF, 20 ng/mL EGF, 5% BSA, 1% penicillin/streptomycin, insulin 5 μg/mL). At day 3, spheres were collected and re-suspended in 200 μL of fresh 15% FBS RPMI with 1% penicillin/streptomycin; spheres were seeded on dried matrigel and incubated for 1 h at 37C, then covered with 3 mL of the same above mentioned medium. Fresh culture medium was added every 3 days.

### Primary cultures

Primary cultures derived from ascites were obtained as described^42, 47, 55^. Cultured cells from borderline tumors were obtained from a pre-surgery peritoneal wash and cultured as described for ascites^42, 47, 55^. Cancer type and stage from patients are summarized in Supplementary Table 1. Ascites samples were centrifuged at 2,500 rpm for 3 min at 25 °C. Cell pellets were resuspended in a 1:1 mixture of ascitic fluid and DMEM-F12 10% FBS and 1% penicillin/streptomycin, 70–80% confluent cultures were used for VM assay. Cultures were used within 2 passages.

### Fluorescent Dye Microinjection

One or 4 day-old cultures were used for Lucifer Yellow Dye (LYD) (Cat. no. L0259-25MG, Sigma, St. Louis, MO) microinjection experiments. Micropipettes were pulled from borosilicate glass capillaries (Cat. N°. 602000, A-M System) and filled with Lucifer yellow (25 mM). Microinjection was performed as described^56^ and images were acquired using an inverted microscope coupled to xenon arc lamp illumination and a Nikon B filter (excitation wavelength 450–490 nm; emission wavelength above 520 nm). For trypan blue microinjection, micropipettes were backfilled with undiluted Trypan Blue (Cat. N°. 15250–061, Gibco Life Technologies, Canada) and connected to a Femtojet microinjector (Cat. N°. 5252000021, Eppendorf, Germany) which applied a constant compensatory pressure of 400 hPa during 20 minutes. After injection time, fluorescent signal corresponding to trypan blue was visualized in a DMI6000 B Leica fluorescence microscope. Images were taken at 5X and visualized using LAS-AF Leica software.

### X-Ray Microtomography (Micro-CT) 3D-Reconstruction

7.500 cells of the SKOV3 cell line were seeded on ¼ (5,5 × 5,5 mm) part of a plastic cover slip (TED PELLA INC. #2225-1, USA) with matrigel as described before. After 4 days on culture VM structures were formed, fixed with PFA 4%, dehydrated with ethanol (increasing concentration from 70 to 100%, 1 hour each) and dried overnight. Samples were then placed in the Micro-CT (Skyscan 1272, Bruker micro-CT, Kontich, Belgium) and scanned with the following settings: 4032 × 2688 camera resolution, 57.43 mm object to source distance, 35KV @ 231 microAmps X-Ray source (no filter), 2.999986 µm pixel size, 1000 ms exposure, 0.15° rotation phase, 360° total rotation, flat field correction. Scan time: 121 minutes. Images were processed using CT Vox 64-bits v.3.1.1 software (Bruker, Belgium).

### Confocal Microscope Imaging

4 day-old 3D-cultures of GFP-HEY cells were fixed and observed in a Zeiss LSM780, confocal microscope system (Center for Advanced Microscopy CMA BIO BIO, Department of Cellular Biology, Faculty of Biological Sciences, Concepción University). Samples were scanned with a Plan ApoChromat NA 1.3 objective using argon laser exposition of 488 nm, EM. 490–560, Z-stack cuts of 80–130 um. For 3D-reconstructions Zen 2012 (Zeiss company) and Imaris 8.4 (Bitplane’s core scientific software) software were used. For PAS fluorescence emission, 4 day-old 3D-Cultures of GFP-HEY were fixed and subsequently stained with PAS as previously described. PAS fluorescence was obtained using an argon laser excitation of 560 nm, EM. 560–630, Z-stack sections were of 80–130 um. For 3D reconstruction, the same programs as listed above were used.

## Electronic supplementary material

Supplementary Information

Supplementary Video 1

Supplementary Video 2

Supplementary Video 3
