# Supplementary material for: Structural and functional identification of vasculogenic mimicry in vitro
Source: Sci Rep. 2017 Aug 1;7:6985. doi: 10.1038/s41598-017-07622-w (PMC5539303; doi:10.1038/s41598-017-07622-w)
Supplement: Supplementary file 1 — Supplementary Information [file 41598_2017_7622_MOESM1_ESM.pdf]

Supplementary Information

Structural and functional identification of vasculogenic mimicry*in vitro*

Authors:
Dusan Racordon[1,*], Andrés Valdivia[1,*],Gabriel Mingo[1],Rafaela Erices[1,2],Raúl Aravena[1,3], Felice Santoro[1], Maria Loreto Bravo[1,4,5], Carolina Ramirez[1], Pamela Gonzalez[1],Alejandra Sandoval[1,6,8], Alfonso González[9,11], Claudio Retamal[10,11], Marcelo Kogan[8], Sumie Kato[2], Mauricio A. Cuello[2], German Osorio[12], Francisco Nualart[12],Pedro Alvares[13], Araceli Gago-Arias[13],Daniella Fabri[13], Ignacio Espinoza[7,13], Beatriz Sanchez[7,13],Alejandro H. Corvalán[1,6,7,8], Mauricio A. Pinto[1] & Gareth I. Owen[1,4,5,6,7,8].

Affiliations:
[1]Department of Physiology, Faculty of Biological Sciences, Pontificia Universidad Católica de Chile, Santiago Chile
[2]Division of Obstetrics & Gynecology, Faculty of Medicine, Pontificia Universidad Católica de Chile, Santiago Chile.
[3]Universidad Santo Tomás, Santiago Chile.
[4]Biomedical Research Consortium of Chile, Santiago Chile.
[5]Millennium Institute on Immunology and Immunotherapy, Pontificia Universidad Católica de Chile, Santiago Chile.
[6]Department of Hematology and Oncology, Facultyof Medicine,Pontificia Universidad Católica de Chile, Santiago Chile.
[7]Center UC Investigation in Oncology at Pontificia Universidad Católica de Chile, Santiago Chile.
[8]Advanced Center for Chronic Diseases (ACCDiS), Facultyof Medicine, Pontificia Universidad Católica de Chile, Santiago Chile.
[9]Facultad de Medicina, Universidad San Sebastián, Santiago Chile
[10]Facultad de Ciencias, Universidad San Sebastián, Santiago Chile
[11]Centro de Envejecimiento y Regeneración, Facultad de Ciencias Biológicas, Pontificia Universidad Católica de Chile
[12]Department of Cellular Biology, Faculty of Biological Sciences, Universidad de Concepcion, Chile.
[13]Institute of Physics, Pontificia Universidad Católica de Chile, Santiago Chile.

*These authors contributed equally to this work

Correspondence:
Gareth I. Owen, Ph.D.,
Tel: (+56)23542854. Fax: (+56) 22225515
Pontificia Universidad Católica de Chile, Facultad de Ciencias Biológicas
Alameda 340, Santiago de Chile,
Postal code: 8331150.
gowen@bio.puc.cl

Contents

1- Supplementary Videos Captions -2-

2- Supplementary Figures and Captions -3-

3- Supplementary Table and Caption -9-

1- Supplementary Videos Captions

Supplementary Video 1

X-ray microtomography (microCT) reconstruction analysis of structures formed in SKOV3 after 4 days in matrigel. Progressive cross sections of the 3D reconstruction show that numerous structures (especially the structure that looks tubular) contain a regions with lower CT intensity values, which indicates the existence of hollow structures (see profile of the relative Hounsfiled Units Supplementary Figure 3).

Supplementary Video 2

IMARIS reconstruction based exclusively on fluorescent cells (i.e. only cancer cells, not any secreted matrix) shows a lumen with fenestrated coating of cancer cells.

Supplementary Video 3

IMARIS reconstruction based exclusively on PAS (stimulated to fluoresce upon laser excitation at 560nm). Red fluorescence represents an elevated glycoprotein-rich layer surrounding the lumen, which is elevated above the cell monolayer (shown in green).

2- Supplementary Figures and Captions

a

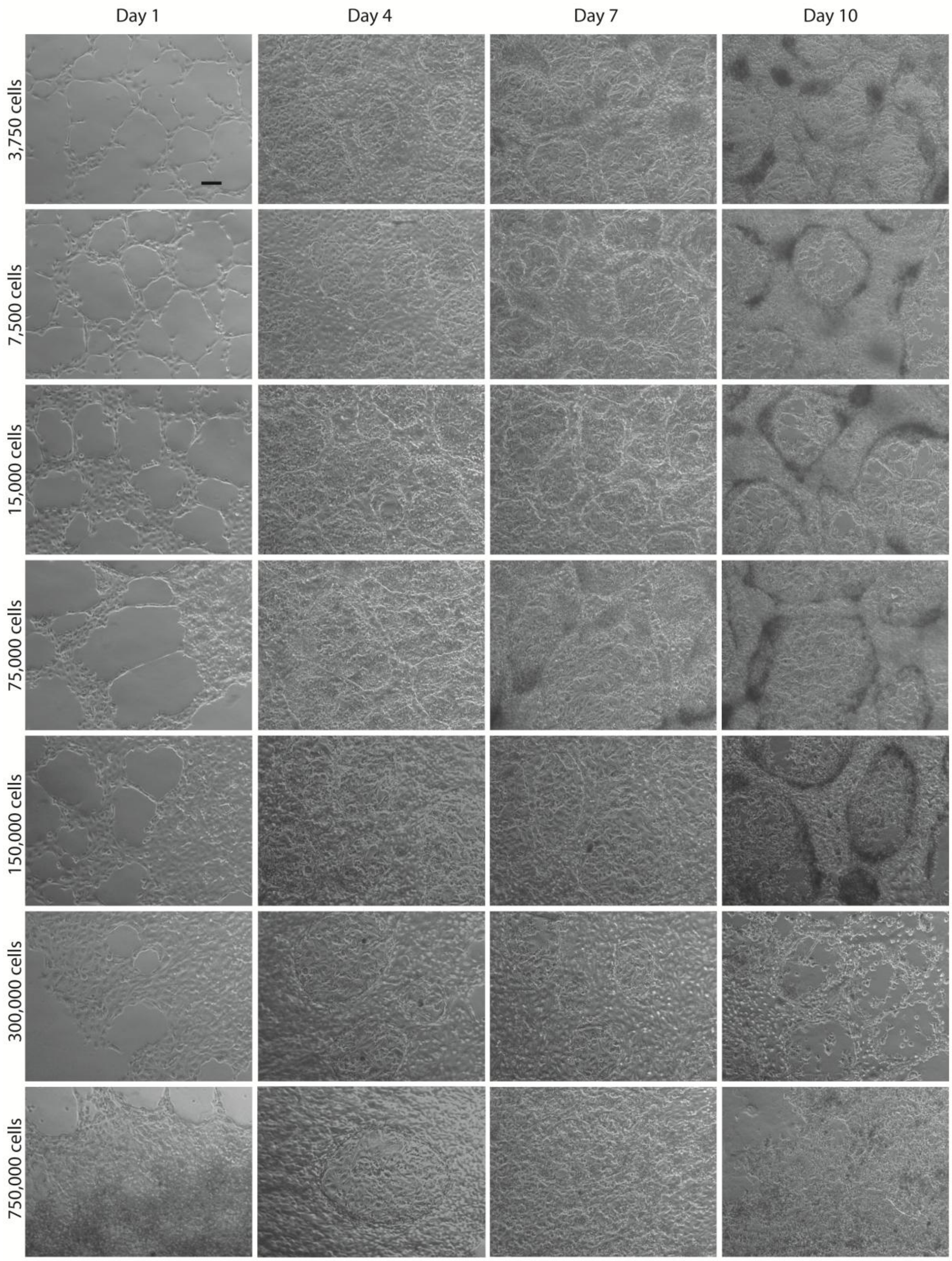

b

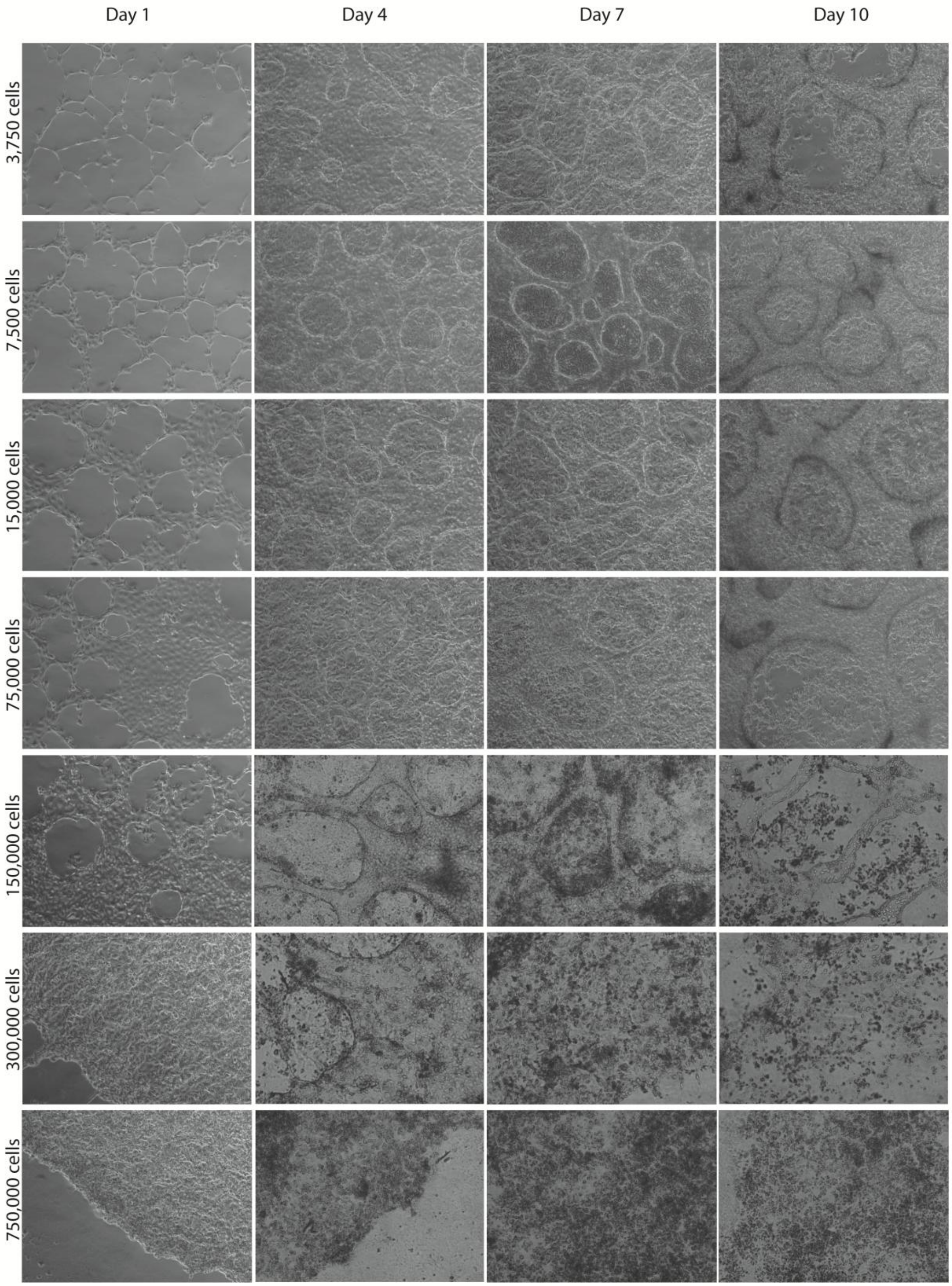
Day 1
Day 4
Day 7
Day 10
3,750 cells
7,500 cells
15,000 cells
75,000 cells
150,000 cells
300,000 cells
750,000 cells

c

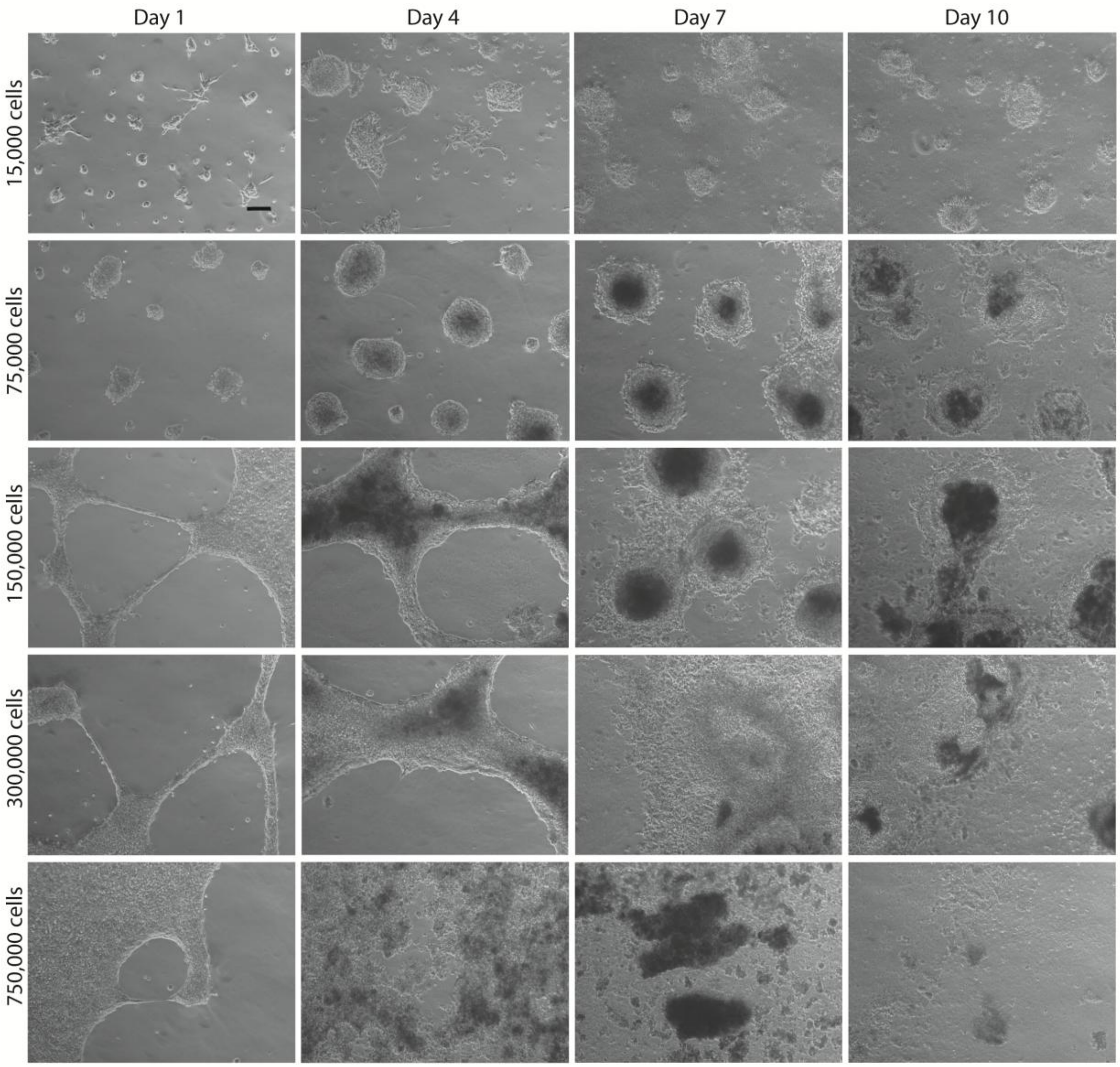

Day 1
Day 4
Day 7
Day 10
15,000 cells
75,000 cells
150,000 cells
300,000 cells
750,000 cells

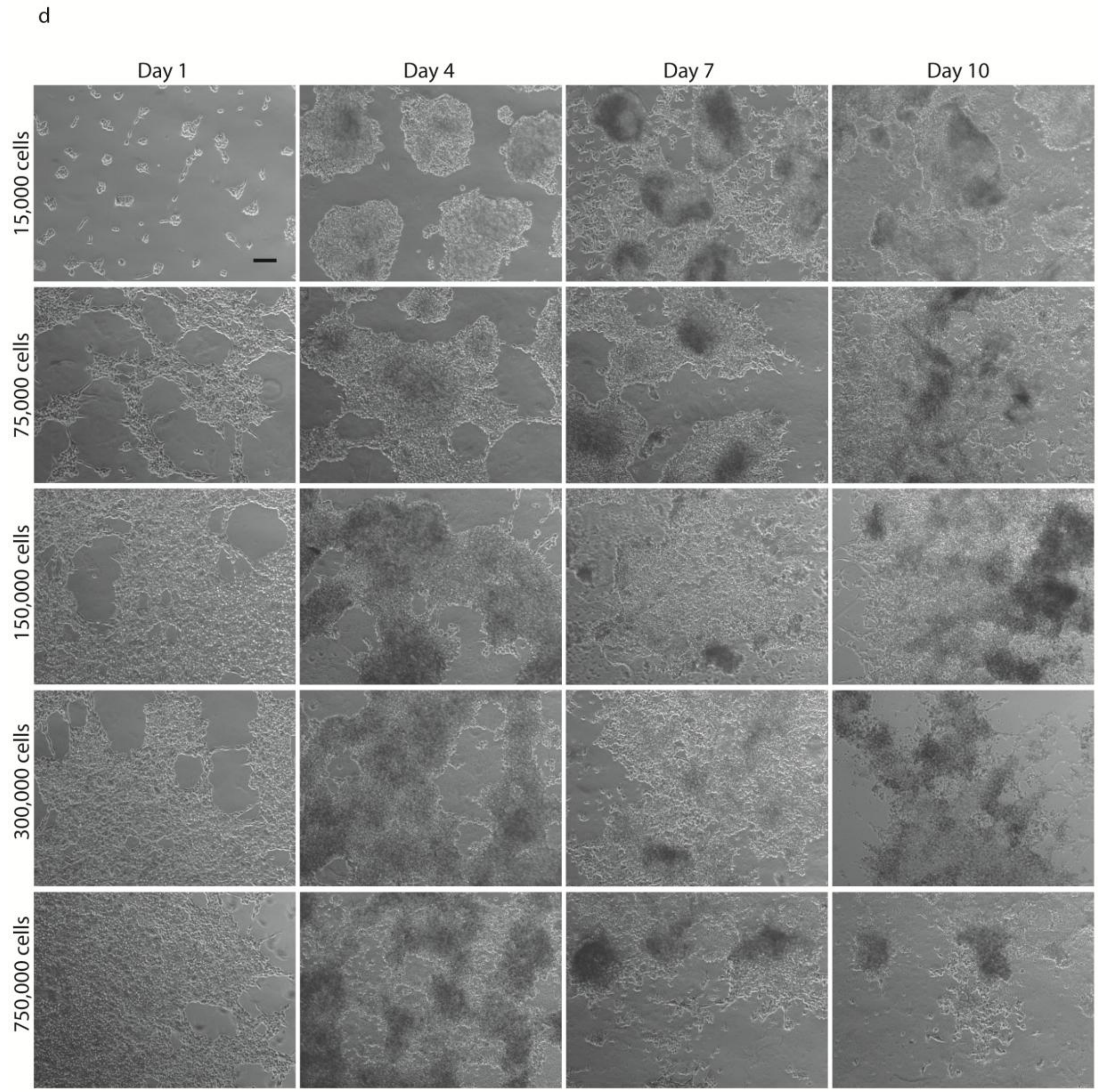

Supplementary Figure 1. Culture time and seeding cell number in the formation of tubular structures in ovarian cancer cell lines. Formation of structures in 3D-cultures on matrigel followed by day (days 1, 4, 7 and 10) in the presence varying seeding cell numbers for SKOV3 (panel a), HEY (panel b), UCI101 (panel c) and A2780 (panel d) cells. Magnification: 10x. Scalebar: 100µm. In both SKOV3 and HEY cultures, seeding with 3.750 or 75000 needed three days longer to reach the stage of tube formation than cultures seeded with 15.000 to 300.000 cells. HEY cultures initiated with 150.000 and above cells started to disintegrate after 4 days in culture. In both SKOV3 and HEY cultures seeded with 750,000 cells the formation of tubular structures initiated briefly at day 4, before being lost. No formation of tubular structures occurred in UCI101 or A2780 cell lines at any time or seeding cell number.

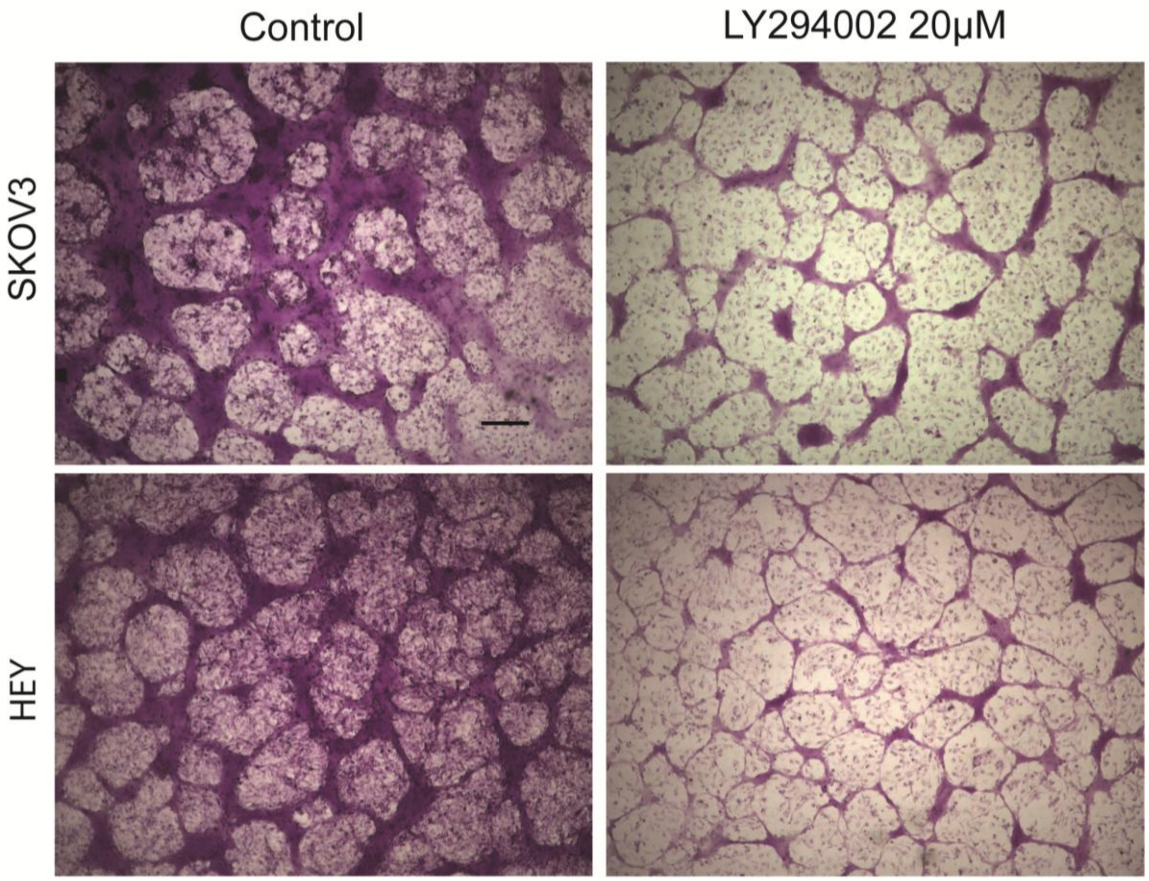

Supplementary Figure 2. Pharmacological inhibition of PI3K reduces tubular structure formation. PAS stained 4 days-old cultures of SKOV3 and HEY cells after daily treatment with LY294002 (20uM). A loss in tubular structures is evident in accordance with previous reports in the literature. Magnification: 4x. Scale bar: 200µm.

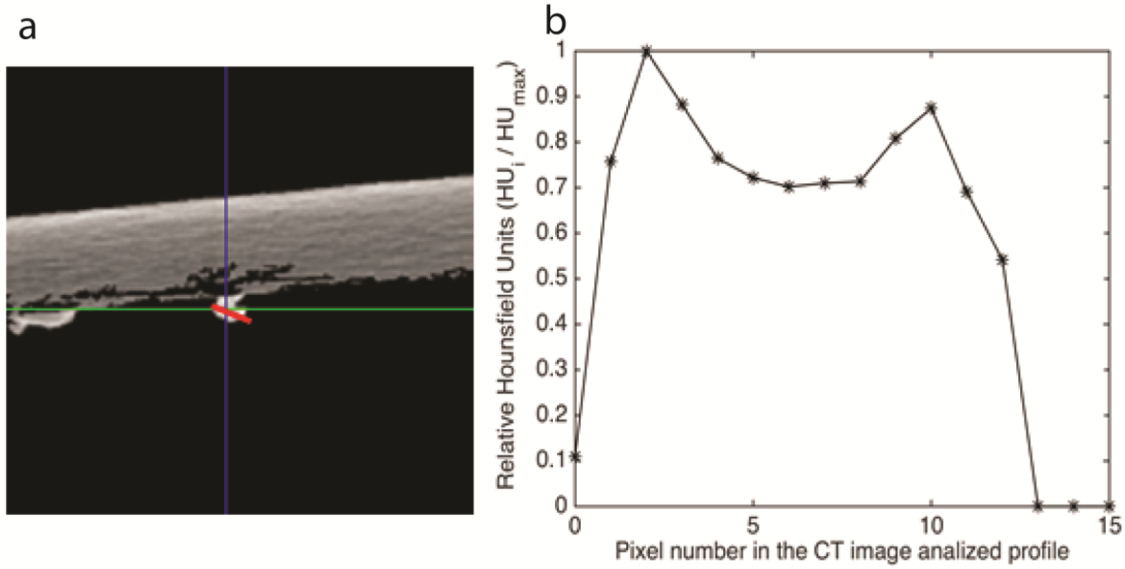

Supplementary Figure 3: Intensity profile of the tubular structures analyzed by microCT. (a) Representative cross-section of tubular structure with a low intensity area in the center. (b) Intensity profile in Hounsfiled Units of the tubular structures studied by microCT. Lower intensity values on the inner region of the tube indicate the presence of a lumen.

3- Supplementary Table and Caption

Supplementary Table 1. Ovarian cancer patient characteristics

Sample	Diagnostic	Hystological Type	Stage	Differentiation Grade	Age (years)	VM
1	Serous	cancer	IIIC	moderate	52	+
2	Serous	cancer	IIIC	moderate	52	-
3	Ovarian fibrothecoma	benign	N/A	N/A	51	-
4	Mucinous	cancer	N/R	well diferentiated	75	+
5	Borderline	borderline	N/A	N/A	63	-
6	Serous	cancer	IIIC	moderate	52	+
7	Ovarian cystoadenoma	benign	N/A	N/A	58	-
8	Ovarian adenocarcinoma	cancer	IIIC	poorly diferentiated	63	+
9	Serous	cancer	IV	poorly diferentiated	48	-
10	Serous	cancer	IIIC	poorly diferentiated	66	-
11	Serous &Endometroid	cancer	IIIB	poorly diferentiated	49	-
12	Granulosa cell tumor	cancer	IA	N/R	54	-
13	Borderline	borderline	N/A	N/A	59	+

Ovarian cancer type, age of patient, stage, histological grade and differentiation as reported in the medical history of the patient. The final column dictates whether the primary cultured cells were capable (+) or not (-) of undergoing vasculogenic mimicry (VM). N/A not applicable, N/R not reported.
